# Hospitals: Their History, Organization and Construction

**Published:** 1877-10

**Authors:** 


HOSPITALS: Their History, Organization, and Construction. Boylston Prize Essay of Harvard University, for 1876. By W. Gill Wylie, M.D. New York: D. Appleton & Co., 1877.

The title of this book is sufficient to create a desire for its perusal. Of the difficulties incident to the proper construction and general management of hospitals few are apprised, and most physicians can yet learn something on these subjects. The principles concerned not only affect the public charge of hospital surgeons, but are more or less concerned in the care of our patients in private practice. The work will, therefore, be found interesting and useful to the general practitioner.

